# Characteristics of Indigenous adults with poorly controlled diabetes in north Queensland: implications for services

**DOI:** 10.1186/s12889-015-1660-2

**Published:** 2015-04-03

**Authors:** David Ross Johnson, Robyn Anne McDermott, Peter Marshall Clifton, Katina D’Onise, Sean Matthew Taylor, Cilla Louise Preece, Barbara Anne Schmidt

**Affiliations:** Aboriginal Health Council of South Australia, 9 King William Road, Adelaide, SA 5061 Australia; College of Public Health, Medical and Veterinary Sciences, James Cook University, Cairns, Australia; School of Pharmacy and Medical Sciences, University of South Australia, Baker IDI Heart and Diabetes Institute, Adelaide, Australia; School of Population Health, University of South Australia, Adelaide, Australia

**Keywords:** Diabetes mellitus type 2, Indigenous, Albuminuria, Risk factors, Primary health care

## Abstract

**Background:**

Indigenous Australian adults with diabetes continue to have suboptimal clinical control and poorer outcomes compared with non-Indigenous people although there is a paucity of data documenting the detailed health status of Indigenous people in Australia. To further investigate the characteristics of Indigenous Australian adults with poorly controlled diabetes we analysed baseline data from a cluster randomized trial aiming to deliver a program of integrated community-based intensive chronic disease management for Indigenous people in remote communities in far north Queensland, Australia.

**Methods:**

Indigenous adults aged 18 to 65 years from 12 clinics in rural north Queensland with established type 2 diabetes and with HbA1c ≥8.5% were invited to participate. The primary outcome variable measured at baseline was HbA1c. Other variables measured included socio-demographic indicators, health literacy, BMI, blood pressure, lipids, renal function, smoking status and quality of life measures. Data were collected between December 2010 and July 2011. Analysis was performed by ethnicity – Aboriginal or Torres Strait Islander.

**Results:**

One hundred and ninety three participants were included in the analysis. Very high rates of albuminuria, high rates of smoking, dyslipidaemia, hypertension and elevated BMI were recorded. Aboriginal participants reported higher levels of socio-economic disadvantage, higher smoking rates, lower BMI and worse self-reported health status than Torres Strait Islander participants.

**Conclusion:**

These results demonstrate a high potential for improved culturally sound community-based management of diabetes and other comorbid conditions in this very high risk population. They also provide further evidence for including albuminuria in cardiovascular risk calculation.

## Background

Indigenous Australians experience very high prevalence, morbidity and mortality from chronic health conditions such as diabetes, cardiovascular, renal and chronic respiratory disease [1-3]. The higher rates of these conditions accounts for the majority of the ‘health gap’ between Indigenous and non-Indigenous Australian’s with those residing in remote areas particularly affected [4]. As a result, Indigenous people experience much higher rates of avoidable hospital admissions from chronic diseases [5,6] and, while the causes of this are complex, a lack of access to effective primary health care services is a major contributing factor [7].

Over recent years there has been an increasing focus both internationally [8] and in Australia [9,10] on enhancing the capacity of primary care delivery systems, originally set up to manage acute disease, to better prevent and manage chronic diseases and their complications. The Chronic Care Model has been influential in enhancing chronic illness care through improving clinical system delivery design, clinical decision support, clinical information systems, patient self-management support, organizational support and mobilizing community resources to better manage chronic disease [8].

At the same time, a number of Indigenous community health services in Australia have undertaken chronic disease quality improvement initiatives adopting many of the Chronic Care model components [11]. While improvements have been made in the provision of routine clinical care processes, there has been less success translating this into improved intermediate clinical outcomes, such as glycaemic control for people with diabetes or reduction in risk factors such as overweight and obesity [12,13]. This has been in part attributed to what has been termed the ‘clinical inertia’ of treating doctors [14], particularly relating to delays in initiating insulin [15]. More importantly, perhaps, has been the under-utilisation of the Indigenous Health workforce. In Australia, there is good evidence that Indigenous Health Workers (IHWs) are well placed to provide routine chronic disease care via the use of disease management protocols which is cost effective as long as there is: (1) adequate training in chronic disease management and health promotion/disease prevention; (2) good clinical support mechanisms and; (3) system-level support to ensure adequate staffing to enable chronic disease self-management support for individuals, families and groups in community settings [16-18].

Recognising the central role played by IHWs, the “Getting Better at Chronic Care” (GBACC) study in North Queensland is a randomized cluster trial that aims to deliver integrated community-based, intensive chronic disease management initially in a small number of Indigenous communities in the Torres Strait, Cape York and Cairns districts in far North Queensland, Australia.

This paper reports on selected baseline clinical and socioeconomic data from this study.

## Methods

### Study design, setting and participant selection

This is a baseline analysis of the GBACC study whose methodology has been described elsewhere [19]. In summary, this is an open parallel randomised cluster trial delivering integrated community-based, intensive chronic disease management, compared to usual care, via rural and remote Indigenous primary health care services to Indigenous Australians living in far North Queensland, Australia. Twelve clinics (clusters) in rural north Queensland were chosen based on having significant Indigenous populations and primary health care services provided either by Queensland Health or a local community-controlled Indigenous Health Service. Potential participants were identified from diabetic disease registers of the participating health services. Adults aged 18 to 65 years from these registers with established type 2 diabetes (diagnosed for more than one year) and with HbA1c ≥8.5% were invited to participate in the study. Written informed consent was obtained from individual participants. Data collection was undertaken between December 2010 and July 2011.

### Variables

The primary outcome variable measured at baseline was HbA1c. Other variables measured included socio-demographic indicators, health literacy, intermediate clinical outcomes other than HbA1c (renal function, blood pressure, lipids), preventative and patient self-care practices and quality of life measures.

Ethnicity was based on self-report. Where participants reported “both Torres Strait Islander and Aboriginal” descent they were classified as Torres Strait Islander for this analysis. Health literacy was measured using the Test Of Functional Health Literacy in Adults (TOFHLA) tool (score 0–100; 0–59 inadequate, 60–74 marginal, 75–100 adequate health literacy) [20]. Renal function was calculated from serum creatinine using estimated Glomerular Filtration Rate (eGFR) calculated by the Chronic Kidney Disease Epidemiology Collaboration (CKD-EPI) formula [21] and albuminuria was defined as a urinary albumin creatinine ratio (ACR) ≥2.5 mg/mmol in men and ≥3.5 mg/mmol in women. Chronic kidney disease (CKD) stage was calculated as per the Kidney Health Australia Caring for Australasians with Renal Impairment (KHA-CARI) guidelines [22]. Those with CKD stage 4 and 5 were excluded from the study. Hypertension was defined as blood pressure >140/90 or if prescribed an antihypertensive medication. Dyslipidaemia was defined as one or more of total cholesterol (TC) ≥4 mmol/L, high-density lipoprotein cholesterol (HDL-C) <1 mmol/L and triglyceride (TG) ≥1.5 mmol/L. Quality of life was measured using the Assessment of Quality of Life (AQoL) instrument and the 36-Item Short-Form (SF-36v2) health survey. The TOFHLA tool, AQoL and SF-36v2 surveys as well as a personal information survey were administered face-to-face with participants. All clinical data was obtained via review of hard copy primary health care charts, patient information management systems (Ferret/Medical Director/Best Practice as applicable) and Auslab/Auscare databases for pathology.

### Ethics approval

The GBACC study was approved by the University of South Australia Human Research Ethics Committee, the University of Queensland Medical Research Ethics Committee and the Cairns and Hinterland Health Service District Human Research Ethics Committee with support from the relevant peak Aboriginal and Torres Strait Islander Health Councils (Trial Registration number 12610000812099). This project has a high level of community engagement through partnering with the local regional Indigenous health organisation, local Indigenous health services and community councils of participating communities. The potential benefits of the study are large both at the individual and family level as well as for the Indigenous health workforce. Further, a major strength of the project is its focus on sustainability which will be achieved by building capability of the local Indigenous health workforce.

### Statistical analyses

Data were analysed by ethnicity as Torres Strait Islanders are genetically, culturally and geographically distinct from mainland Aboriginal Australians. We were interested in exploring clinical as well as socio-demographic differences between these populations which may have relevance to diabetes/chronic disease management. Chi square tests were used to compare proportions, student’s t-tests for comparison of categorical and normally distributed continuous variables, Kruskal-Wallis tests for comparison of categorical and non-normally distributed continuous variables and Spearman Rank correlation tests for comparison of non-normally distributed continuous variables. Linear regression was also performed to further assess associations. All analyses were undertaken using Stata version 11 (Stata Corp, TX, USA).

## Results

One hundred and ninety three participants who met the modified inclusion criteria were included in the baseline data analysis. This represented approximately 75% of the eligible population on registers who were invited to participate. Equal numbers of Torres Strait Islander (n = 97) and Aboriginal people (n = 96) were represented.

### Socio-demographic characteristics for Aboriginal and Torres Strait Islander participants

The mean age for participants was 48 years. Overall there were a higher proportion of female participants (64%) and this was most marked for Aboriginal people (70%) (Table 1).

**Table 1:** **Socio-demographic indicators by ethnicity, chronic care study, 2011 (N = 193†)**

	**Aboriginal**	**Torres Strait Islander**	**Overall**	**P-value**
**Number of participants**	**96**	**97**	**193**	
Mean (95% CI) age (years)	47.7 (45.9-49.6)	47.5 (45.5-49.5)	47.6 (46.2-49.0)	0.92
Number (%) of women	67 (70)	56 (58)	123 (64)	0.08
Employment status (%)				0.003
*Full-time*	25 (26)	41 (42)	66 (34)	
*Part-time*	14 (15)	22 (23)	36 (19)	
*Unemployed*	57 (59)	34 (35)	91 (47)	
Education level attained (%)				<0.001
*Did not complete year 12*	77 (80)	52 (54)	129 (67)	
*Year 12 certificate*	2 (2)	16 (16)	18 (9)	
*TAFE course*	14 (15)	19 (20)	33 (17)	
*University course*	3 (3)	10 (10)	13 (7)	
Median (interquartile range) income ($)	15080 (11960–27690)	28600 (14300–36400)	18980 (13000–33800)	<0.001
Not enough money for food (%)	49 (51)	25 (26)	74 (38)	<0.001
No. of missed meals past fortnight (%)				<0.001
*None*	49 (55)	93 (98)	142 (77)	
*One to two*	26 (29)	1 (1)	27 (15)	
*Three or more*	14 (16)	1 (1)	15 (8)	
Median (interquartile range) TOFHLA score	86.2 (72.5-94.0)	86.2 (68.8-91.1)	86.2 (71.1-93.0)	0.23
No. of people per house (interquartile range)	5 (3–7)	4 (3–7)	4 (3–7)	0.84
Smoking				
*Current smoking (%)*	43 (46)	33 (35)	76 (41)	0.13‡
*Never smoked (%)*	33 (35)	37 (40)	70 (38)	
*Ex-smoker (%)*	17 (18)	23 (25)	40 (21)	
*Current female smoker (%)*	33 (50)	20 (36)	53 (44)	
*Current male smoker (%)*	10 (37)	13 (34)	23 (35)	
Mean (95% CI) body mass index (kg/m2)	30.3 (28.6-32.0)	34.2 (32.2-36.2)	31.9 (30.6-33.3)	0.004

†Missing data: Missed meals 9, Smoking 7, BMI 106.

‡Smoker/non-smoker as binary.

A high number of participants were not currently employed (47%) although it was lower for Torres Strait Islanders (35%) than for Aboriginal people (59%) (p = 0.003). Aboriginal participants were also less likely to have completed year 12 (p = <0.001), reported lower median incomes (p = <0.001), were more likely to not have enough money for food at times (p < 0.001) and were more likely to have missed meals in the previous fortnight (p < 0.001) compared to Torres Strait Islanders (Table 1).

Despite this, there was little difference in median TOFHLA scores by ethnicity but they were higher for those who had completed year 12 (8.3, 95% CI, 2.6 to 13.9, p = 0.004) after controlling for age, sex and ethnicity*.*

Overall the proportion of smokers was high (41%) with Aboriginal people more likely to smoke (46%) compared to Torres Strait Islanders (35%) (Table 1). Most of the difference in smoking prevalence was accounted for by a higher proportion of female Aboriginal smokers. Torres Strait Islander mean body mass index (BMI) scores were much higher than for Aboriginal people (BMI score 4.3, 95% CI, 1.8 to 6.7, p = 0.001) after controlling for smoking, age and sex.

### Intermediate clinical outcomes and preventive care practices

Even though only participants with HbA1c ≥8.5% were included, median HbA1c overall was still very high (10.9%) (Table 2). There was a weak association observed between lower HbA1c and higher TOFHLA score although this was unlikely to be clinically important (for a 10 point increase in TOFHLA score HbA1c decreased by 0.23%, 95% CI, −0.49 to +0.029%, p = 0.082).

**Table 2:** **Baseline intermediate clinical outcomes, chronic care study, 2011 (N = 193†)**

	**Aboriginal**	**Torres Strait Islander**	**Overall**	**P-value**
Median (interquartile range) HbA1c	10.9 (9.6-12.1)	11.0 (9.7-12.4)	10.9 (9.7-12.2)	0.38
Hypertension diagnosed (%)	75 (78)	71 (74)	146 (76)	0.50
Mean (95% CI) systolic BP (mmHg)	131.0 (126.9-135.2)	131.6 (127.6-135.7)	131.3 (128.5-134.2)	0.84
Mean (95% CI) diastolic BP (mmHg)	80.0 (77.8-82.3)	78.8 (76.5-81.1)	79.4 (77.8-81.0)	0.44
Hypertension diagnosed and normal BP‡ (%)	38 (51)	39 (55)	77 (53)	0.61
Albuminuria (%)	56 (76)	47 (73)	103 (75)	0.76
Median (interquartile range) estimated glomerular filtration rate (ml/min/1.73 m^2^)	103.9 (88.6-111.4)	94.7 (77.1-115.7)	100.6 (80.9-113.0)	0.27
Chronic kidney disease stage				0.08
*Normal renal function*	17 (24)	19 (29)	36 (27)	
*Stage 1 (%)*	35 (49)	21 (32)	56 (41)	
*Stage 2 (%)*	15 (21)	14 (22)	29 (21)	
*Stage 3 (%)*	4 (6)	11 (17)	15 (11)	
*Total*	71	65	136	
Mean (95% CI) total cholesterol (mmol/L)	4.7 (4.4-5.0)	4.6 (4.3-4.9)	4.6 (4.4-4.8)	0.53
Total cholesterol <4 mmol/L (%)	25 (31)	27 (38)	52 (34)	0.38
Mean (95% CI) high-density lipoprotein cholesterol (mmol/L)	0.90 (0.85-0.95)	1.05 (0.77-1.3)	0.98 (0.83-1.12)	0.32
High-density lipoprotein cholesterol ≥1 mmol/L (%)	25 (38)	18 (26)	43 (32)	0.13
Mean total cholesterol: HDL ratio (95% CI)	5.3 (4.9-5.7)	5.3 (4.9-5.7)	5.3 (5.0-5.6)	0.88
Median (interquartile range) triglyceride (mmol/L)	2.2 (1.5-3.5)	1.7 (1.1-2.8)	2.0 (1.2-3.1)	0.02
Triglyceride <1.5 mmol/L (%)	19 (24)	33 (46)	52 (35)	0.004
Dyslipidaemia (%)	78 (98)	66 (93)	144 (95)	0.19
Taking insulin (%)	35 (36)	43 (44)	78 (40)	0.27
Monitors own blood sugar (%)	45 (47)	37 (39)	82 (43)	0.22
Adherent to all medications (%)	46 (48)	31 (32)	77 (40)	0.02
Prescribed anti-hypertensive medication if diagnosis of hypertension (%)	67 (89)	67 (94)	134 (92)	0.27
Albuminuria and prescribed renin-angiotensin inhibitors (%)	45 (80)	42 (89)	87 (85)	0.13
Prescribed lipid lowering medication (%)	77 (80)	68 (70)	145 (75)	0.10

†Missing data: Systolic and Diastolic BP 9, Hypertension diagnosed 1, Albuminuria 55, CKD 54 (3 CKD stage 4 not included), TC 42, HDL-C 59, TG 43, Dyslipidaemia 42, monitors blood sugar 2, sometimes miss medication 9.

‡Defined as <140/90.

A high proportion were diagnosed with hypertension and, although overall mean systolic and diastolic readings suggested reasonable blood pressure control, nearly half of those with a diagnosis of hypertension recorded blood pressure readings >140/90 .

The proportion of those with albuminuria was extremely high (75%) and the majority of participants were classified as having stage 1 to stage 3 chronic kidney disease (73%) with CKD stage being slightly less advanced for Aboriginal compared to Torres Strait Islander participants. Overall, mean high-density lipoprotein cholesterol (HDL-C) was low (0.98 mmol/L) with only 32% of participants recording normal HDL levels. Mean total cholesterol: HDL-C ratios were also high (5.3).

Forty per cent of participants were prescribed insulin and 43% monitored their own blood sugars.

The majority of those with a diagnosis of hypertension were prescribed an anti-hypertensive medication (92%) and relatively high proportions of those with both albuminuria and dyslipidaemia were presribed renin-angiotensin inhibitors (angiotensin converting enzyme inhibitors and/or angiotensin receptor blockers) (85%) and lipid lowering medication (75%) respectively. Torres Strait Islander participants were less likely to be adherent to all medications (p = 0.02) (Table 2). Insulin use was not associated with medication adherence.

### Selected quality of life and self-reported health measures

Although Aboriginal participants were less likely to be anxious or depressed, they reported more pain and tiredness and they were less likely to report excellent or very good health compared to Torres Strait Islanders (Table 3).

**Table 3:** **Quality of life measures by ethnicity, chronic care study, 2011 (N = 193)**

	**Aboriginal**	**Torres Strait Islander**	**Overall**	**P-value**
**Anxious or depressed**				<0.001
*No*	37 (39)	18 (19)	55 (29)	
*Slightly*	47 (49)	75 (77)	122 (63)	
*Moderately*	12 (12)	4 (4)	16 (8)	
**Pain**				0.01
*None*	58 (60)	79 (81)	137 (71)	
*Moderate*	31 (32)	15 (16)	46 (24)	
*Severe*	5 (5)	3 (3)	8 (4)	
*Unbearable*	2 (2)	0	2 (1)	
**Self-reported health status**				0.008
*Excellent or very good*	12 (13)	30 (31)	42 (22)	
*Good*	50 (52)	41 (42)	91 (47)	
*Fair or poor*	34 (35)	26 (27)	60 (31)	
**Tired**				<0.001
*All or most of the time*	20 (21)	6 (6)	26 (13)	
*Some of the time*	50 (52)	38 (39)	88 (46)	
*A little or none of the time*	26 (27)	53 (55)	79 (41)	

Anxious or depressed Q14 from the Assessment of Quality of Life Survey (AQoL); pain Q15 from AQoL survey; self-reported health status Q1 from SF-36v2 Health Survey; tired Q9i from SF-36v2.

## Discussion

This Indigenous population with poorly controlled diabetes recorded very high rates of albuminuria, a non-traditional cardiovascular risk factor, as well as high rates of smoking, elevated BMI, dyslipidaemia and hypertension. Differences related to ethnicity included higher levels of socio-economic disadvantage, higher smoking rates, lower BMI and worse self-reported health status for Aboriginal people compared to Torres Strait Islander people.

The prevalence of albuminuria (77%) was very high for this group of Indigenous people. High rates of albuminuria have previously been reported in Indigenous populations in Australia with and without diabetes [23-26] most pronounced in remote locations [27] and at much higher levels than for non-Indigenous Australians [28,29]. In our study, despite the higher prevalence of albuminuria, median eGFR was still relatively high although our results are limited by the exclusion of those with CKD stage 4 and 5. Disproportionately high rates of albuminuria compared to eGFR have previously been reported in Indigenous populations [26,29] which may reflect different age profiles and rates of diabetic or obesity related hyper-filtration compared to non-Indigenous populations. Albuminuria may also be a better indicator of progression of CKD than eGFR in those with type 2 diabetes or it may reflect differences in the origins and pathophysiology of CKD in Indigenous versus non-Indigenous populations [26,30]. For our study eGFR was calculated using the CKD-EPI formula which provides better estimates compared to the MDRD formula [31].

There is good evidence that albuminuria independently predicts renal and cardiovascular risk in diabetics [32,33] which is the leading cause of death for diabetics. Albuminuria can be reduced through the use of renin-angiotensin inhibitors [32,34]. In this Indigenous population 85% of those with albuminuria were prescribed a renin-angiotensin inhibitor. This is higher than the rate of renin-angiotensin blockade in an urban sample of both Indigenous Australians with diabetes (47%) and non-Indigenous Australians with diabetes (66%) [29]. However, it may be that despite the appropriate drugs being prescribed, the dose titration may not be adequate to impact most effectively for renal protection.

Only 40% of Indigenous participants were prescribed insulin despite the high glycaemic levels. This may still reflect a degree of clinical inertia [35] on the part of medical staff working with Indigenous clients, as they are not being treated as intensively as clinically indicated.

Although previous studies have shown inadequate health literacy is associated with worse glycaemic control [36,37], there was little evidence for such a relationship for Indigenous participants in our study. It may be that there was not the diversity of HbA1c measures to show such a relationship given that only people with uncontrolled diabetes were included. It was also interesting to note that despite the relative socioeconomic disadvantage, including lower year 12 completion rates, Aboriginal people had similar health literacy scores to Torres Strait Islanders. This suggests either limitations with the TOFHLA tool used in this setting or that other unmeasured factors were compensating for this disadvantage.

Women were over-represented in the Indigenous sample of people with uncontrolled diabetes. This has previously been shown in both remote and urban Indigenous Australian populations [38] as a result of higher rates of obesity in Indigenous women placing them at higher risk of diabetes [39]. This is in contrast to non-Indigenous populations where women and men are either equally represented [29,38] or men over-represented [40]. In our sample, BMI was significantly lower for Aboriginal compared to Torres Strait Islander participants and this persisted after controlling for age, smoking status and sex. Such differences have previously been reported [28] and may be explained by a greater relative lean body mass for given height for Torres Strait Islanders given their genetically Melanesian origins [41,42]. Relative food insecurity as a result of relative socio-economic disadvantage for Aboriginal participants may also explain some of this difference in our study.

The mean age of people in our sample was very young. It has previously been shown that Indigenous Australians are diagnosed with diabetes at a much younger age than non-Indigenous Australians [28,29]. Possible explanations include a survival bias whereby Indigenous populations experience more risk factors (such as obesity and smoking) for diabetes and diabetes related complications resulting in higher mortality rates in the middle age years [3] as well as environmental influences in early life such as poor intrauterine growth [43] leading to earlier and more progressive disease.

There were limitations to this study. Indigenous participants were recruited from communities who in turn were selected based on the likelihood that their health service had the capacity and the organizational support to deliver the intervention and so results may not be generalizable to all Aboriginal and Torres Strait Islander communities. Although interesting, our results related to health literacy should be treated with caution. Current assessment tools, including the TOFHLA survey, essentially measure reading skills and may not differentiate between reading ability and background knowledge of scientific health concepts and/or cultural differences in the understanding of health and health care [44]. Further, given that only people with diabetes and HbA1c levels ≥8.5% were included, the results may not be generalizable to all Indigenous Australians with diabetes. For some clinical variables there were missing data although the characteristics of participants with missing data were similar to those with data recorded. This pragmatic trial in a highly mobile population reflects the realities of delivering optimal diabetes care. Prevalence of albuminuria may be overestimated as urinary ACR calculations were based on a single sample and microscopy, culture and sensitivity was not performed at the time of collection.

Effective and sustained delivery of diabetes care by Indigenous Health Workers who are linguistically and culturally close to their clients has previously been demonstrated in the Torres Strait [12,16,45]. This resulted in much improved continuity of care compared to that provided by an itinerant and often inexperienced non-Indigenous workforce. It is our view that better utilisation of the Indigenous health workforce has the potential to increase the health standards in high need populations through the delivery of more culturally safe health care and greater engagement with the extended family of people living with diabetes.

## Conclusion

As such, these results demonstrate a high potential for improved culturally-sound integrated community-based management of diabetes and other comorbid conditions in this very high risk population. They also provide further evidence for including albuminuria in cardiovascular risk calculation in clinical settings in addition to traditional risk factors [46].
